# Promoting Advance Care Planning for Persons with Dementia: Study Protocol for the LEAD (Life-Planning in Early Alzheimer’s and Other Dementias) Clinical Trial

**DOI:** 10.21926/obm.icm.2301004

**Published:** 2023-01-06

**Authors:** Kara B. Dassel, Eli Iacob, Rebecca L. Utz, Katherine P. Supiano, Hollie Fuhrmann

**Affiliations:** 1. University of Utah, College of Nursing, 10 S. 2000 E., Salt Lake City, UT, USA; 2. University of Utah, College of Social and Behavioral Sciences, 260 South Central Campus Drive, Salt Lake City, UT, USA

**Keywords:** Decision-making, care partner, persons with dementia, goals of care

## Abstract

Due to the insidious progression of Alzheimer’s disease and related dementias (ADRD), surrogate decision-makers typically make medical and long-term-care decisions for a care recipient, most often a family care partner. Unfortunately, many care recipient/care partner dyads have failed to engage in advance care planning or have lost the opportunity to do so due to the cognitive decline of the care recipient. To address this need, our team created a validated dementia-focused advance care planning tool known as the LEAD Guide (Life-Planning in Early Alzheimer’s and Other Dementias). With funding from the National Alzheimer’s Association and in consultation with our community advisory board, we developed a preliminary web-based intervention. This intervention integrates the LEAD Guide with self-paced educational modules that lead dyads through conversations and dementia-focused advance care planning processes. In this concept paper, we describe the aims of our funded R01 clinical trial (National Institute on Aging), where we aim to refine our preliminary web-based platform for use in a 5-month mixed-method NIH Stage-1 behavioral intervention. Using a sample of diverse community-based ADRD dyads (n = 60), we aim to: 1) describe the acceptability, usability, and feasibility of the intervention, 2) assess the initial efficacy of the intervention on the primary outcome (decision-making self-efficacy), and secondary outcomes (relationship quality, subjective well-being, anxiety) as perceived by both the care recipient and the care partner, and 3) examine advance care planning congruence as a mechanism of action. The LEAD clinical trial addresses public health challenges by guiding and supporting families through challenging advance care planning conversations, facilitating the transfer of knowledge regarding care preferences and values from the care recipient to the care partner, with the ultimate goal of improving the quality of life for both individuals with ADRD and their care partners.

## 1. Introduction

Preparation for end-of-life, in terms of advance care planning, is a significant area of interest for both clinicians and researchers, especially as our population ages [1–4]. Advance care planning is a communication process that empowers adults of any age and any state of health to articulate and share their values, life goals, and preferences regarding future medical care [5]. Advance care planning is essential for persons with Alzheimer’s disease or related dementias (ADRD), as end-of-life healthcare often relies on the substituted judgment of family care partners [6, 7] after the care recipient loses decision-making abilities [8–10].

Persons with ADRD are less likely to complete an advance directive [11, 12] appoint a surrogate decision-maker [13, 14], or engage in advance care planning conversations with family [15]. They are, therefore at higher risk of unnecessary medical interventions, frequent hospital readmissions, numerous transitions between healthcare settings [16, 17], and higher end-of-life healthcare costs [7, 18] compared with their cognitively intact peers. Furthermore, care partners who have not engaged in advance care planning with the care recipient report feeling unprepared and burdened in their role as surrogate decision-makers [19–22]. This feeling of unpreparedness is not surprising given that the care partner’s knowledge and accuracy of the care recipient’s care preferences are less than 50% accurate [23]. Conversely, dyads that engage in advance care planning have been shown to have lower anxiety [24, 25], higher levels of subjective well-being [24, 26], greater decision-making self-efficacy [27–30] (the dyad’s confidence that the care partner can make informed decisions that align with the care recipient’s values and preferences), and greater advance care planning congruence (a shared understanding of the care recipient’s care values and preferences) [22, 31–33].

Advance care planning involves both formal documentation (completion of an advance directive) and informal communication (ongoing conversations with family and healthcare providers about care values and preferences) [5]. Research has shown that advance care planning is most effective when care recipients complete formal documents after conversing with future surrogate decision-makers [34]. However, approximately only half of adults engage in comprehensive advance care planning [35, 36], with even lower rates among those who have never married, are of diverse racial and ethnic backgrounds, are of low socioeconomic status, and have an ADRD diagnosis [36–38]. In the context of ADRD, communication and transfer of knowledge from the care recipient to the care partner [39–42] are critically important, as the care partner will inevitably become involved in decision-making when the care recipient’s cognitive deterioration advances [43, 44]. Therefore, persons with ADRD must engage in formal and informal advance care planning to achieve value-congruent medical care at the end of life [42, 45–47].

Many existing tools guide patients and their families through the advance care planning process. However, the design of these tools is for use in specific healthcare settings, such as primary care [15, 48–51], nursing homes [14, 52–56], and hospitals [45, 57, 58]. Many require a third party to facilitate [50, 51, 59], are not theoretically developed or psychometrically validated [60–62], and are paper-based tools that do promote engagement in meaningful conversations [59, 63, 64]. Furthermore, no existing tools focus specifically on preparing care partners to gain the knowledge and competence [39, 41, 65–67] they need to confidently make care and treatment decisions that will emerge across the ADRD trajectory [24, 68]. In addition, existing research has primarily excluded persons with ADRD, assuming they cannot cognitively participate in the advance care planning process [24, 69–74]. Recent research has found that persons in the early stage of ADRD can engage in advance care planning conversations [75–77]. However, such conversations ought to be started as early as possible, before the loss of decision-making abilities [61, 78].

To address these gaps, our team developed a dementia-focused advance care planning tool called the LEAD Guide (LEAD stands for Life-Planning in Early Alzheimer’s and Other Dementias) [79, 80]. The LEAD Guide addresses changes in cognition and goals of care along the ADRD continuum. The guide is value-based rather than focused only on documenting specific medical decisions [75, 78, 81]. The guide also anticipates the need for a surrogate decision-maker upon loss of the care recipient’s decisional abilities [7, 78, 82]. The LEAD Guide is the first dementia-focused advance care planning tool created using established instrument-development procedures to determine psychometric validity and reliability [83, 84]. However, like many of the existing advance care planning interventions, the LEAD Guide is a paper-based tool with limited reach and little opportunity for care recipients and their families to (re)engage in advance care planning after its initial completion.

We first received funding from the National Alzheimer’s Association to use the LEAD Guide to develop a pilot advance care planning intervention. We then received funding from the National Institute on Aging (R01) to refine our pilot intervention for implementation in a national web-based dementia advance care planning clinical trial. The remainder of this manuscript outlines the R01 LEAD clinical trial protocol.

## 2. Materials and Methods

### 2.1 Study Overview

We will conduct a 5-month mixed-method NIH Stage-1 [85] behavioral intervention to evaluate the feasibility and initial efficacy of the LEAD clinical trial. We will first refine the LEAD clinical trial based on our pilot work (Stage 1A) and then evaluate its usability, acceptability, feasibility, and initial efficacy (Stage 1B) in a diverse sample (including advance care planning under utilizers) [36–38] community-based ADRD dyads (spousal/partner or parent/child, n = 60) who have yet to engage in advance care planning.

The LEAD clinical trial addresses the limitations of existing advance care planning interventions in that it is web-based [63, 86], self-administered [50, 51, 59], and focused on a community-based population [15, 51, 52, 58]. This web-based application will integrate the LEAD Guide with self-paced educational modules that guide dyads through the process of dementia-focused advance care planning. We expect to show that the LEAD clinical trial will improve outcomes related to decision-making self-efficacy through greater advance care planning congruence. In addition, we anticipate that this intervention will improve subjective well-being, anxiety, and relationship quality as perceived and reported by both the care recipient and the care partner.

#### 2.1.1 Research Aims and Study Hypotheses

The LEAD clinical trial aims to improve decision-making self-efficacy by increasing advance care planning congruence within dyads. Specifically, the LEAD clinical trial aims to facilitate the transmission of knowledge from the care recipient to the care partner by providing advance care planning education, resources, and hands-on activities. These activities provide a space where the care recipient and the care partner [87] can engage and are encouraged to re-engage in conversations about the care recipient’s care values and preferences across the various stages of dementia.

**Figure 1:**
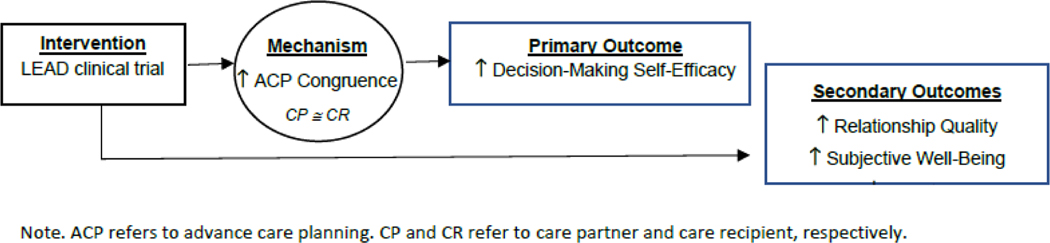
Conceptual model of intervention effect.

We focus on three specific aims with hypotheses: 1) Aim 1. To describe the acceptability, usability, and feasibility of the LEAD clinical trial. 2) Aim 2. To assess the initial efficacy of the LEAD clinical trial on the primary outcome (decision-making self-efficacy) and secondary outcomes (relationship quality, subjective well-being, anxiety) as perceived by both the care recipient and the care partner. 3) Aim 3. To examine advance care planning congruence as a mechanism of action for the LEAD clinical trial. As shown in Figure 1, advance care planning congruence, as facilitated by the dyad participating in the LEAD clinical trial, is hypothesized to be the mechanism of action that underlies the trial’s effect. Specifically, this mechanism enhances advance care planning decision-making self-efficacy, as perceived by both the care recipient and the care partner.

### 2.2 Recruitment and Retention

We will use a convenience sample of 60 study dyads (120 persons) to evaluate the LEAD clinical trial. We are explicitly looking for persons in the preclinical or early stage of cognitive decline who have not previously engaged in advance care planning. Older adults with subjective cognitive complaints and those with mild cognitive impairment are at increased risk of progressing to dementia compared with their cognitively normal counterparts [88–93]. Persons beyond the early stage of ADRD may find challenges in the tasks required for data collection and intervention participation [94–96]. In contrast, those who have yet to engage in advance care planning can receive coaching on how to engage in comprehensive advance care planning [60, 64, 97, 98]. In order to broaden recruitment opportunities and because this is a community-based (rather than clinic-based) study, we will not administer any cognitive screening assessments or verify self-reported diagnoses.

Drawing from our prior experience [79–80], we will use three national research registries for recruitment: ResearchMatch^©^, TrialMatch^©^, and Clinical Trials^©^. ResearchMatch^©^ is a national health-research volunteer research registry supported by the U.S. National Institutes of Health as part of the Clinical Translational Science Award program. TrialMatch^©^ is a free matching service supported by the National Alzheimer’s Association that connects individuals with ADRD, care partners, and healthy volunteers to authorized clinical research studies. Clinical Trials^©^ is a National Institutes of Health registry of publicly and privately funded clinical trials. To preferentially recruit communities that have historically been less likely to adopt advance care planning (racially/ethnically diverse groups, LGBTQ individuals) [36–38], we will engage in University-affiliated and community-based recruitment strategies.

#### 2.2.1 Consent Procedures

**Figure 2:**
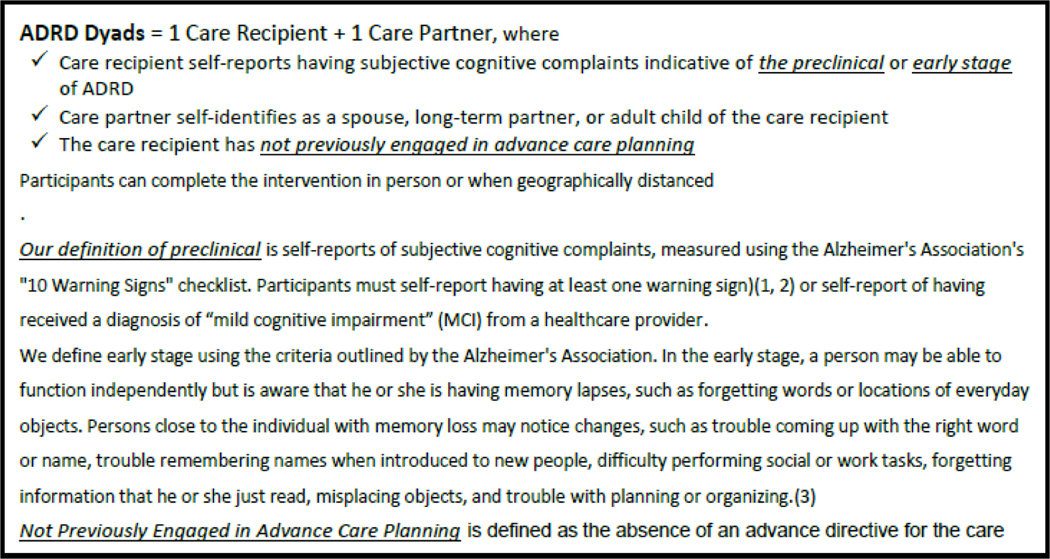
Participant inclusion criteria.

The study received approval from the University of Utah Institutional Review Board (IRB# 00132042). We will invite potential dyads who meet inclusion criteria to participate in the study (See Figure 2).

For these individuals, a research team member will contact the potential dyad by phone at a time convenient to both potential participants. Before the phone call, the research team member will send the potential participants an electronic copy of the informed consent document. During the phone call, the research team member will review the consent document, including a description of voluntary participation, study procedures, risks, and potential benefits, and answer any questions. The research team recognizes that potential participants with preclinical or early-stage ADRD may need additional time or explanation to make an informed and voluntary decision regarding consent. As needed, the research team member will schedule a follow-up phone call.

We will implement an “understanding of consent protocol” consistent with standards outlined in The National Bioethics Advisory Committee Belmont Report [99] and Research Involving Persons with Mental Disorders that May Affect Decision-Making Capacity Report [100]. Participants will respond to several standardized questions concerning the study procedures and participation rights to ensure comprehension before giving consent. While this is a minimal-risk study, and participants will not be vulnerable in the narrow definition of the Belmont Report, we recognize that persons with early memory loss may have potential vulnerabilities that we must respect. We further recognize the importance of including persons with early memory loss in research, as required by federal law (42 USC 289a-2) and NIH policy (NOT-OD-18–014 and NOT-OD-18–116). Dyads who consent and respond in a manner that confirms they understand the study procedures and consent process will enroll in the study.

### 2.3 Intervention

#### 2.3.1 The LEAD Guide

The LEAD Guide is the foundation of this advance care planning clinical trial. The LEAD Guide includes three sections; review of documentation, end-of-life values, and end-of-life preferences. The documentation section asks the participant three questions: 1) whether they have completed the documentation for a living will or advance directive, 2) whether they have completed a do not resuscitate order or DNR, and 3) whether they have completed the necessary documentation to appoint a medical power of attorney and if so, to name the individual.

The following section focuses on end-of-life values. This section describes values as the participant’s attitudes towards end-of-life care that typically do not change much and, therefore, can serve as guideposts for the care partner as they make decisions on their behalf. The first set of questions asks participants to rate their level of agreement with statements about their level of concern about being a financial, emotional, and physical burden to their family or friends. The following section asks participants to rate their agreement with statements about how they weigh the quality of life and length of life concerning their future end-of-life care. The final section in the values portion of the LEAD Guide asks participants to describe whom they would like to be involved in decision-making about their end-of-life care (i.e., the patient, family, healthcare provider, or combination thereof). There is a supplemental area at the end of the values section to write additional information about their values.

The final section of the LEAD Guide focuses on end-of-life care preferences, described as malleable depending on one’s circumstances. The participants complete a series of statements about their preferences for end-of-life care in two distinct situations, based on their current abilities to articulate their preferences and in a hypothetical future scenario where they will require their care partner to make decisions on their behalf. Participants state their preferred location for receiving 24-hour supervision and care (i.e., at home, in someone else’s home, in a residential hospice center, in a nursing home, in a hospital, or uncertain). The following section asks participants about their preferences for receiving or not receiving life-prolonging care, such as a breathing tube, medical interventions if their brain had stopped working, and feeding through a tube. The last section asks about preferences regarding controlling the timing of one’s death concerning voluntarily not eating or drinking, self-directed means, or legally receiving medications from a physician to hasten death. There is a supplemental area at the end of the values section to write additional information about their preferences for care now or in the future.

The LEAD Guide also includes a glossary that defines technical terms such as advance directive, do not resuscitate order, and end-of-life. When creating the LEAD Guide, we gave special attention to health literacy, and as such, the LEAD Guide is at an eleventh-grade reading level. Lastly, there are additional pages at the end of the guide for participants to write any additional information regarding their end-of-life wishes, such as how they define “quality of life.”

#### 2.3.2 Best Care Practice Recommendations

The LEAD clinical trial implements best-practice recommendations for ADRD clinical trials and advance care planning research and practice [24, 70, 101]. As such, the LEAD clinical trial has several unique attributes. First, the LEAD clinical trial is a multicomponent intervention, providing education and hands-on activities to guide persons with ADRD and their care partners through the various tasks of advance care planning. Multicomponent caregiver interventions, meaning that they included both education and active participation (i.e., applying knowledge and skills), have positive long-term effects on care partner outcomes such as burden, depression, and subjective well-being. In contrast, information-only interventions had little to no effect on most care partner outcomes [70, 101, 102]. Similarly, advance care planning interventions that focused only on written educational materials are less effective than those that combined written materials with opportunities for skills training, education, and interactive discussions [69, 73, 103]. Multicomponent caregiver interventions lower care recipients’ risk of institutionalization and reduce problematic behavioral symptoms [70, 104].

Second, the LEAD clinical trial is a web-based intervention. Although not all persons will feel comfortable using technology-delivered applications, a web-based platform has notable advantages, including fewer barriers associated with training, billing, and timing [63, 69, 71, 105]. Web-based delivery allows for self-administration and access to persons in rural areas. Note that tablets with enabled cellular service are available to participants in rural areas that may need access to technology and internet service. In addition, this delivery method includes care partners unable to leave care recipients due to a lack of respite options and for caregivers who are geographically distanced [106, 107]. Web-based delivery is feasible, effective, cost-effective, scalable, and easy to implement [63, 107–109]. In addition, such interventions do not rely on trained third-party facilitators and allow participants the convenience to do them at their own pace in the privacy of their own homes [110]. A web-based advance care planning intervention does not take the place of such conversations with healthcare providers. However, it can help prepare care recipients and care partners for such conversations. A review of 11 web-based advance care planning interventions (e.g., Death over Dinner [111], Five Wishes [112], Making Your Wishes Known [113], PREPARE for Your Care [114] showed improved advance care planning knowledge, communication, and documentation [63]. However, we are aware that no web-based intervention focused on advance care planning in the context of dementia [86].

Third, the LEAD clinical trial is a dementia-focused intervention. People likely have different anticipated care values and preferences based on their understanding of the illness trajectory [80]. In contrast to other common illnesses and causes of death, the ADRD illness trajectory is tied to one’s cognitive functioning and eventual loss of decisional abilities, requiring persons with ADRD to rely on care partners to make decisions on their behalf. Thus, it is critical for persons with ADRD to have ongoing advance care planning conversations with a trusted care partner and complete formal advance care planning documents. The LEAD clinical trial aims to help the care recipient and the care partner develop a shared understanding (i.e., advance care planning congruence). This congruence allows them to feel confident (i.e., decision-making self-efficacy) that future care decisions will be informed and guided by the care recipient’s values and preferences. Recent reviews and meta-analyses found that only 10 out of 167 advance care planning studies and interventions focused on the specific needs of ADRD [24, 63, 69, 115]. As the National Alzheimer’s Association and the U.S. Department of Health and Human Services increase their efforts to promote early diagnosis of dementia [116, 117], the LEAD clinical trial can serve as a beneficial resource to initiate the advance care planning process within the ADRD population.

Fourth, the LEAD clinical trial benefits the care recipient and the care partner. Although advance care planning, by definition, is focused on the documentation of a care recipient’s values and preferences for end-of-life care, to achieve advance care planning, there must be communication and sharing with their families and care partners. This process is especially true in the context of ADRD, where a transfer of information from the care recipient to the care partner is typically required, given the loss of care recipient autonomy associated with the progression of dementia over time. When advance care planning is comprehensive, care partners will gain a well-informed understanding of the care recipients’ values, preferences, and specific healthcare wishes (congruence). By improving congruence and decreasing uncertainty among the care recipient and care partner [46, 63, 118, 119], the surrogate decision-making burden will be reduced [120]. Care partners will feel empowered to make future decisions on behalf of the care recipient. Care recipients will feel more confident (decision-making self-efficacy) that future healthcare decisions align with their values and preferences. As a secondary outcome, the care recipient and care partner may feel less anxious and have more positive subjective well-being due to participating in the LEAD clinical trial. Discussion of advance care planning needs and concerns [68] may enhance the dyad relationship quality. This dual-focused feature of the intervention is in line with the ADRD clinical trial recommendation to include caregiver outcomes in intervention studies [121], which historically have focused almost exclusively on care recipients’ needs and health experiences.

Lastly, only a few previous studies have explored the causal mechanisms underlying the efficacy of advance care planning interventions or tracked outcomes following intervention completion (i.e., three months or beyond) [86]. Most were conducted in specialized clinical settings, focused solely on the care partner or care recipient (not both), and did not use a standardized method to measure congruence [69, 122]. Our proposed community-based study, utilizing a longitudinal, mixed-method design, addresses these gaps and will provide rich data on the process and outcomes associated with advance care planning in an ADRD population.

#### 2.3.3 Intervention Design

The LEAD clinical trial is intended to be self-administered and delivered through a comprehensive, interactive, web-based platform designed according to recommended functionalities and user-designed principles [63]. Through three distinct modules (See Table 1), the LEAD clinical trial will facilitate the advance care planning processes of 1) defining the care recipient’s care values and preferences, 2) developing advance care planning congruence within the dyad, or a shared understanding of the care recipient’s values and preferences, through conversation(s), and 3) encouraging ongoing advance care planning conversation and sharing of documentation beyond the dyad. These processes are the foundation of Module 1: Individual, Module 2: Together, and Module 3: Documentation and Sharing.

**Figure 3:**
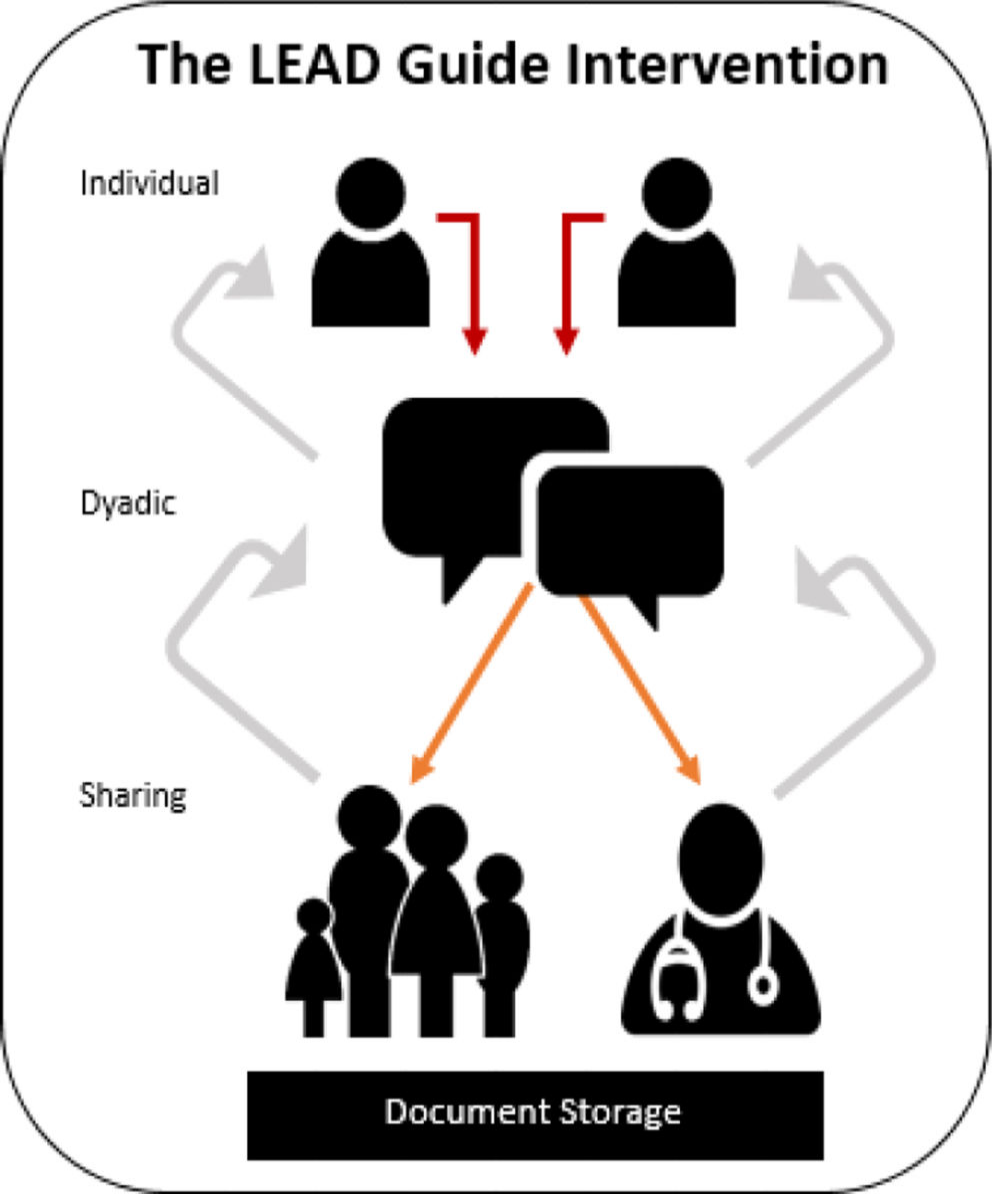
Intervention model.

All modules will include video tutorials introducing the goals and tasks of each module, as well as interactive resources relevant to the content of each module (e.g., review of dementia progression, explanation of life-sustaining treatment options, communication techniques for difficult conversations) [63]. In Module 1, each dyad member completes the LEAD Guide. The care recipient completes based on his/her values and preferences for their end-of-life care, and the care partner completes the LEAD Guide based on how they believe the care recipient will respond to each question. In Module 2, the dyad will video-record themselves, utilizing a built-in video-recording software, comparing their responses on the LEAD Guide as they identify areas of congruence and discordance. Following their conversation, they will complete the LEAD Guide together if revisions are needed based on their conversation. In Module 3, participants are to revise and save formal advance care planning documents and record additional conversations, creating a web-accessible archive of conversations and thoughts about advance care planning values and preferences [63]. The three modules follow a sequential pattern, each prompted one week apart, giving participants time to learn, reflect, and complete the tasks. As shown with light-gray arrows in Figure 3, the dyad may revisit any of the three modules during the 5-month study period [63].

**Figure 4:**
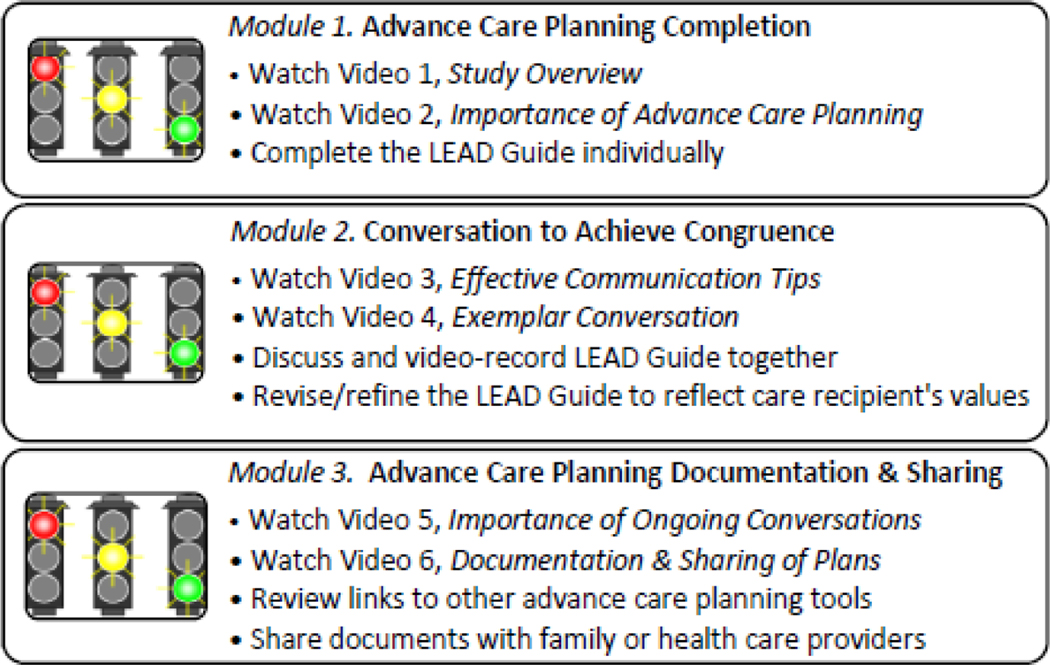
Dashboard tracking intervention completion.

For eventual real-world implementation, the dyad would have access to the three modules, including the document-storage/sharing functions, allowing them to revisit advance care planning as the disease progresses or conditions change. Participants will see a dashboard similar to Figure 4, which tracks completing the recommended tasks associated with each intervention module. The dashboard indicates which tasks have been or still need to be completed [63]. All participants will receive weekly auto-generated messages (text or e-mail, based on communication preference at enrollment) reminding them of each module’s tasks, resources, and tools.

#### 2.3.4 Benefits for Participants and Researchers

A web-based intervention will provide several advantages for the study participants and the study team [63]. For study participants, a web-based intervention will 1) create a convenient, home-based, self-administered intervention program, 2) allow study participants the opportunity to save and electronically disseminate their completed advance care planning documents to extended family members and healthcare providers, 3) serve as a personal archive for video files of advance care planning conversations, and 4) provide easy access to the research team for comments, questions, or concerns. For the research team, a web-based intervention will afford 1) the creation of informational videos on how to engage in challenging advance care planning conversations as well as providing an exemplar discussion, 2) integration of data collection with intervention procedures by allowing participants to video-record conversations and document other real-time information about their end-of-life values and preferences, 3) the ability to auto-send information and personalized reminders to participants to increase treatment fidelity and participant adherence, 4) reduced study costs by minimizing the need for trained facilitators and by minimizing missing data and participant attrition given the ease of intervention completion in one’s own home and at one’s convenience, and 5) potential for national recruitment of research participants, given that dyads can access the LEAD clinical trial from anywhere.

### 2.4 Research Design

#### 2.4.1 Timeline

Our study procedures have two distinct stages, which correspond to the Stage 1A and Stage 1B activities outlined by the NIH stages of behavioral intervention development and testing [85]. In Stage 1A, we will refine the LEAD clinical trial as a web-based intervention, using a collaborative and iterative process consisting of technical development, feedback, refinement, and user testing. In Stage 1B, we will conduct a mixed-method evaluation of the web-based LEAD clinical trial to understand the process and outcomes of advance care planning in diverse community-based ADRD dyads. Together, these two stages will refine and evaluate the web-based intervention’s feasibility, usability, and initial efficacy. We will use the RE-AIM Framework [123] as a general orienting framework to guide our evaluation. RE-AIM, standing for Reach, Efficacy, Adoption, Implementation, and Maintenance, will serve to identify the essential elements that will help us create an evidence-based intervention that is effective, generalizable, and ultimately scalable to widespread use and adoption in the real world.

In Stage 1A (Years 1–2), we will collaborate with our community advisory board and the Genetic Science and Learning Center at the University of Utah in the development, prototyping, and refinement of the LEAD clinical trial as a comprehensive, interactive, and user-friendly platform for older adults and others with cognitive impairment [63, 107–109, 124, 125]. The Genetic Science and Learning Center team includes software engineers, programmers, graphic designers, media producers, and project managers who create web-based applications for healthcare interventions. Our community advisory board will provide feedback at least four times during the intervention-development process, primarily focusing on the imagery, language, and user instructions that are most relevant and easy to use for participants. The media team will produce a series of informational and instructional videos for the web-based intervention platform using a similar collaborative and iterative process used to receive feedback from the board.

In Stage 1B (Years 2–5), we will evaluate the web-based LEAD clinical trial with a sample of community-based ADRD dyads. We will use an integrated mixed-method study design to address three Specific Aims, corresponding to 1) the acceptability, usability, and feasibility of the LEAD clinical trial, 2) the initial efficacy of the LEAD clinical trial on the primary outcome (decision-making self-efficacy) and secondary outcomes (relationship quality, subjective well-being, anxiety), and 3) exploring advance care planning congruence as a mechanism of action for the LEAD clinical trial.

#### 2.4.2 Procedures

Upon consent and enrollment, each dyad member will receive a username and password that will grant them access to the LEAD clinical trial web-based platform, where they will first complete a baseline assessment. The intervention modules, post-intervention, and follow-up assessments will be sequentially unlocked when the previous step is complete. The baseline survey, as well as the mid-intervention (Week 3), post-intervention (Week 8), and follow-up assessments (Weeks 14 and 20), provide both quantitative scores and open-ended narrative responses for key variables. This longitudinal study design will provide a total of five repeated measures of outcome variables; decision-making self-efficacy [126], relationship quality [127], anxiety [128], and well-being [129]. See Table 2 for details.

Participants will access the LEAD clinical trial immediately after completing the baseline survey and will have four weeks to complete the intervention tasks. Additional data will be collected directly from the LEAD clinical trial website. This data includes video recordings of advance care planning conversations (Module 2). In addition, data includes a series of usability, acceptability, and feasibility measures collected within and after the completion of each module (Modules 1, 2, 3). Lastly, Google Analytics will track end-user statistics, such as participant navigation and utilization of the LEAD clinical trial features.

#### 2.4.3 Intervention Fidelity and Data Collection

Guided by the well-regarded RE-AIM framework [123], our proposed mixed-method study design will explore the Reach, Efficacy, Adoption, Implementation, and Maintenance of the LEAD clinical trial. The RE-AIM framework will guide the development and evaluation of this behavioral intervention. The LEAD clinical trial platform will integrate study design (i.e., consent and data collection) and intervention delivery into a unified system, allowing the investigators to continually and systematically manage and track enrollment, consent, intervention completion, and data capture using standard metrics.

The sample is fully powered to get parameter estimates for the feasibility, usability, acceptability, and initial efficacy of the LEAD clinical trial. The proposed mixed-method study will allow us to evaluate study hypotheses while exploring the proposed mechanism of action underlying the intervention’s effect. The LEAD Guide is the foundation of the LEAD clinical trial; it is a published and publicly accessible advance care planning tool that will promote reproducibility. We plan to share all data and resources following the University of Utah and NIH policies. Our data-sharing plan includes storing data in the National Alzheimer’s Coordinating Center, a National Institutes of Health-funded repository.

### 2.5 Measures and Data Collection

#### 2.5.1 Primary and Secondary Outcomes

Different data sources and measures will explore each of the three specific aims.

##### Aim 1:

To describe the acceptability, usability, and feasibility of the LEAD clinical trial, we will use data from the web-based feasibility surveys conducted immediately after the completion of Modules 1, 2, and 3, as well as analytics tracked in the back-end database of the intervention.

To assess feasibility, we will use measures including the time required to identify and recruit 60 dyads, the percent of the enrollment of eligible participants (60/100, or 60%), and retention rate and data completion (85% retention at the end of the intervention; 75% retention to 14-week follow-up; 70% through 20-week follow-up).

To assess the acceptability, feasibility, and appropriateness of the LEAD clinical trial, we will use a validated scale developed by Weiner et al. [130] (Cronbach’s α = 0.85 to 0.91). Each domain includes four Likert-type questions from 1 (Completely Disagree) to 5 (Completely Agree). Our benchmark for success will be a mean of the four items ≥ to 4 on each domain. We will ask a final open-ended question, “Do you have any comments or suggestions for the LEAD clinical trial?” for intervention refinement.

The usability of the LEAD clinical trial is related to end-user behaviors using Google Analytics to describe participant navigation and utilization of the LEAD clinical trial features. These data will describe, for example, which features and resources are utilized the most by participants. These data will also track which participants created, stored, or shared advance care planning documents within the functionality of Module 3.

##### Aim 2:

To assess the initial efficacy of the LEAD clinical trial on the primary (decision-making self-efficacy) and secondary outcomes (relationship quality, subjective well-being, anxiety) as perceived by both the care recipient and the care partner. Table 3 presents the quantitative measures for the primary and secondary outcomes.

##### Aim 3:

To examine advance care planning congruence as a mechanism of action for the LEAD clinical trial. During Module 1, members of the dyad will complete the Lead Guide individually. The care recipient completes the guide for him- or herself, and the care partner completes it as if they were the care recipient. The LEAD Guide questions examining congruence are composed of two types: end-of-life values and preferences using the care recipient’s preferences/values as the “gold standard.” The first category is questions related to end-of-life values on a Likert-type, 1 to 5 scale; for example, “I am concerned about being a financial burden to family or close friends,“ rated from Strongly Disagree to Strongly Agree. Congruence between dyads is the absolute difference between the care partner and the care recipient on a question (e.g., the care partner endorses strongly agree (score of 5) while the care recipient endorses agree (score of 4), leading to an absolute difference of one unit). Reverse scoring each difference will make it such that summing across all questions of interest, higher scores indicate higher congruence.

The second category is end-of-life preferences; for example, “I prefer that decisions related to the location of ongoing care be made by” the care recipient alone, the care recipient with family and doctor, family/or doctor without the care recipient’s input, or uncertain. These will be coded between individuals as either Agree or Disagree. Again, higher scores would be indicative of more congruence. Descriptive statistics will summarize congruence during baseline. In Module 2, the dyad will complete the LEAD Guide together and engage in further advance care planning discussion. Qualitative assessment of advance care planning congruence is a positively weighted change toward a shared understanding of the care recipient’s end-of-life values and preferences. We collect additional indicators via surveys (collected at post-intervention and 20-week follow-up).

Finally, the baseline assessment includes participant demographics. These include age, race, ethnicity, gender, education level, income, comorbid health conditions, and the dyad relationship type. Charlson Comorbidity Index (CCI) [131], collected on the baseline survey, measures comorbidities. The CCI contains 19 health conditions (e.g., diabetes, renal disease), each weighted according to their potential influence on mortality. Each item is assigned a score of 1, 2, 3, or 6 based on the risk of mortality associated with each condition (range = 0–33, with higher scores indicating a greater risk of mortality).

#### 2.5.2 Analytic Plan

A strength of using a mixed-method approach for the LEAD clinical trial is that it will allow us to triangulate measures to generate a comprehensive understanding of the LEAD clinical trial processes and outcomes. For Aim 1, we will systematically track and descriptively report variations using the LEAD clinical trial. Feasibility will be operationalized based on time to enrollment, survey completion, and design-related retention rate. Furthermore, we will implement the three-domain validated Weiner Scale described above to assess each module’s acceptability, feasibility, and appropriateness. We will also use responses from questions on the self-report surveys, utilizing both Likert-type responses and narrative text boxes. We will assess variation in the dosing (completion of modules and how many times module(s) needed to be revisited prior to completion) of intervention delivery. Dose outcomes will be reported separately for each module as the proportion of dyads that completed an advance directive and disseminated advance care planning documents by the end of the study. Dropouts are non-completers under an intention-to-treat analysis.

We will focus on the analysis of data video-recorded through the use of the LEAD clinical trial (dyad conversation in Module 2). These data will be entered into MAXQDA^©^ software and evaluated using qualitative conversation analysis [132, 133]. Conversation analysis utilizes existing dialogue practices and underlying normative organizations of interactions within dyads and the analysis of patterns exhibited across collections of cases. Conversation analysis permits the identification and interpretation of both action and sequence of conversation elements (turn-taking, repair, action formation, ascription, and action sequencing). These elements illuminate the process of shared progress toward an outcome (completion of the LEAD Guide and other outcome measures) [134, 135] (See Table 3 for details).

For Aim 2, we will assess the initial efficacy of the primary (decision-making self-efficacy) and secondary (relationship quality, anxiety, subjective well-being) outcomes for the dyads enrolled in the study. Because individuals are distinguishable (care recipient and care partner) and nested in pairs, bivariate response models are utilized to model the dyad for changes in outcomes across the study. We will utilize time as a categorical variable to allow more flexibility in examining evolution in the primary and secondary outcomes across the five-time points of baseline, midpoint, post-intervention, and 14-week and 20-week follow-ups. This process allows for separate estimation of fixed effects of means for the care recipient and the care partner and allows random variation for the baseline intercepts. Finally, because repeated measures are correlated, and measures between individuals nested in pairs are likely to be correlated, the models will include a covariance matrix that allows for these associations. Our alternative hypothesis is that mean decision-making self-efficacy and secondary outcomes will improve in the care recipient and care partner over time. Additional exploratory covariates will include the dose (number of completed modules), relationship type (spousal/partner versus child-parent), and care recipient gender.

Qualitative descriptive analysis [41, 42] identifies and analyzes themes from open-ended narrative responses associated with these outcome measures. This method emphasizes a “data-near” approach, accepting the respondent’s statement as an accurate representation of their experience.

For Aim 3, qualitative analysis creates profiles of dyads under different categories of both practical and dyad outcomes: ADRD status (preclinical or early stage ADRD), relationship type (spouse/partner or adult child), and education level (of the care recipient). Conversation analysis coding and interpretation will incorporate and expand on themes identified earlier using qualitative descriptive analysis. Coding will also explicate and interpret individual and dyadic responses to the use of the LEAD clinical trial, specifically the process of change in advance care planning congruence. The analysis will further illuminate the relationship quality of dyads and the decision-making self-efficacy of the care partner, providing innovative triangulation of data obtained from self-reported measures and providing a deeper and richer analysis for this aim.

Conversation analysis, coupled with descriptive qualitative analysis (of other narrative responses reported on surveys) and descriptive quantitative analysis (of scaled scores reported on surveys), will illuminate the change process in advance care planning congruence. Furthermore, analyses will illuminate change within the dyad as they move toward outcomes such as advance directive completion and dissemination of documents. We expect that some dyads will begin as highly congruent in their understanding of the care recipient’s goals of care in the context of dementia. Other dyads will proceed toward congruence as a function of the intervention. Still, others may remain incongruent despite the LEAD clinical trial. We will qualitatively explore these difficulties with respect to relationship quality, barriers within the LEAD clinical trial, and dyad type (preclinical vs. early-stage ADRD). Together, these results will provide insight into what types of dyads (e.g., poor relationship quality, higher level of cognitive impairment) might need facilitated support when using the LEAD clinical trial.

Quantitative analysis will explore the role of advance care planning congruence (scoring as described above) on decision self-efficacy in two ways: 1) Hypothesis testing that mean self-efficacy at baseline as well as collapsed over time will be positively associated with advance care planning congruence between the care partner and care recipient at baseline. 2) Describe how congruence at baseline predicts change in self-efficacy for both the care partner and care recipient. This sub-aim is interested in characterizing potential patterns of self-efficacy in dyads based on their initial congruence. We will graphically examine trajectories for patterns from our 60 dyads as well as estimate slopes of self-efficacy for potential interactions with congruence. For example, steep rates of improvement for dyads that start with low congruence and low self-efficacy that complete all modules compared with those with high congruence and baseline self-efficacy may have shallower slopes.

#### 2.5.3 Sample Size

The sample has ample statistical power for the proposed analyses. For example, Aim 1 evaluates feasibility, acceptability, and usability. With a total sample of 60 dyads, we expect reasonable descriptive parameter estimates and narrow 95% confidence intervals for all quantitatively assessed indicators. For example, 60% enrollment starting from a population of 100 dyads has an estimated 95% CI of [50.2%, 69.8%]. Similarly, if we anticipate that 85% of participants complete the surveys post-intervention, a sample size of 60 dyads at baseline has an estimate and 95% CI of 85% [76%, 94%].

Aim 2 examines the initial efficacy of the LEAD clinical trial on primary and secondary outcomes over time utilizing bivariate response models. Power and sensitivity analysis were computed in MPLUS and SAS using a bivariate model with time as a categorical variable and accounting for repeated measures and nesting of dyads. For this study, we will assume differences between 0.2 and 0.4 SD for the care recipient and care partner over time. With 42 dyads (the number of dyads expected to complete all three modules), this gives us greater than 91% power to detect these effects for dyads with complete data. Because our bivariate model analyses use intention-to-treat data from all participants regardless of missing time points, this provides a conservative power calculation for our expected sample size of 60 dyads.

Analyses for Aim 3 are composed of both qualitative and quantitative sub-aims. The proposed study is powered to address Specific Aims 1 and 2 with 60 participant dyads. Aim 3 is descriptive, focusing on mean differences in decision-making self-efficacy based on advance care planning congruence and exploring patterns of change over time based on baseline congruence.

### 2.6 Discussion

Our study is innovative because it utilizes the psychometrically validated LEAD Guide, developed by our team, as a basis for the intervention [79]. We used our preliminary data to guide the concept, design, and implementation of dementia-focused advance care planning protocols with diverse local groups, national samples of healthy adults, adults in the preclinical or early stage of ADRD, and current and former care partners in community-based settings. The LEAD clinical trial is also innovative because it applies to early cognitive decline stages. In addition, the LEAD clinical trial likely has utility for healthy adults and those at risk of or concerned about ADRD but not yet experiencing any symptoms related to cognitive decline. Previous research has provided ample evidence that advance care planning, particularly within the context of ADRD, can reduce unnecessary and unwanted healthcare utilization (e.g., hospitalization, feeding tube) and reduce the stress associated with making surrogate medical decisions at the end of life [56, 136].

The LEAD Guide can be used in clinical settings by healthcare providers to help guide difficult conversations about current and future care associated with progressive neurodegenerative diseases such as ADRD. Given the challenge of engaging in end-of-life planning conversations, the LEAD Guide can help focus the conversation and ensure that the patient’s values and preferences are discussed and documented for future reference.

The LEAD clinical trial design encourages care recipients and care partners to revisit and revise advance care planning as cognitive impairment worsens, the caregiving context changes, or complicating medical conditions arise. Thus, because it explicitly guides dyads to consider whether and how care values and preferences differ under these different types of scenarios, the LEAD clinical trial could be used as a general, comprehensive advance care planning tool. Lastly, the LEAD clinical trial is innovative in implementing ADRD caregiver and advance care planning intervention best-practice recommendations. Furthermore, it is the first advance care planning intervention that is dementia-focused, web-based, self-administered, and intended for community-based settings.

#### 2.6.1 Trial Status

We anticipate to begin enrollment into the LEAD clinical trial in the first quarter of 2024.

## Figures and Tables

**Table 1:** Intervention components.

Module 1: Individual	The care partner and care recipient complete the LEAD Guide individually. The care recipient will complete it based on their values and preferences; the care partner will complete it based on what they believe to be the care recipient’s values and preferences. Comparing the individual LEAD Guides will measure advance care planning congruence at baseline.
Module 2: Together	The care partner and care recipient video-record themselves having a conversation, which focuses on developing a shared understanding of the care recipient’s advance care planning values and preferences. Dyads can record a single or multiple conversation(s) at this stage. All video recordings will be managed and stored by in-app features and functionality. We will use the conversation analyses of recorded conversations to describe the process of how dyads achieve advance care planning congruence.
Module 3: Documentation and Sharing	The care partner and care recipient document and share the advance care planning documentation with other family members and healthcare providers. In-app features and functionality will encourage the creation of additional video diaries to document advance care planning values and preferences. We provide education and resources to create formal advance care planning forms (e.g., advance directive, medical power of attorney, last will and testament, physician orders for life-sustaining treatment, and to facilitate sharing through direct e-mail/share functions and creation of printable PDFs of the LEAD Guide and other advance care planning documents.

**Table 2:** Measures of key outcome variables.

Decision-Making Self-Efficacy	Timing	References
26-item scale with two 13-item scenarios asking whether a family member feels confident to make decisions for another family member who is ill, focusing on whether the care recipient is conscious or unconscious. Care recipient scale will be slightly modified to reflect the confidence that the care partner can decide on their behalf.	W1 W3 W8 W14 W20	Family Decision-Making Self-Efficacy Scale [126]

**Relationship Quality**		

10-item scale asking relationship questions regarding interactions over the past month, such as anger, depression, resentment, and patience. Will be reported by the care recipient and care partner.	W1 W3 W8 W14 W20	Dyadic Relationship Scale (DRS) [127]

**Anxiety**		

8-item self-report questionnaire (e.g., “I felt anxious” or “My worries overwhelmed me”) on a 5-point Likert-type scale (1 = Never to 5 = Always) covering the previous 7 days. Scores range from 7 to 35, with higher scores indicating greater severity of anxiety.	W1 W3 W8 W14 W20	PROMIS Emotional Distress-Anxiety–Short Form 8a [128]

**Subjective Well-Being**		

5-item questionnaire about general life satisfaction (e.g., “I am satisfied with my life”) using a 7-point Likert-type scale (1 = Strongly Disagree to 7 = Strongly Agree). Scores range from 5 to 35, with higher scores indicating greater satisfaction.	W1 W3 W8 W14 W20	PROMIS General Life Satisfaction–Short Form 5a [129]

**Table 3:** Elements of conversational analysis.

Definition and Concept	Conceptual Definition	Evidence of Advance Care Planning Congruence (Presence or Absence of)	Relationship Quality (Presence or Absence of)
Turn-Taking	Alternating who is speaking to “hear each other”	Listening; leads to understanding	Respect, mutual regard vs. impatience
Repair	Acknowledges misunderstanding and self-corrects	Willingness to “give and take” to gain a shared understanding	Respect vs. strain, resentment
Action Formation	Initiation of statement to partner	The assumed topic of discussion and discussion norms	Agreement on the subject and scope of discussion
Action Ascription	Attributing intent to the partner’s statement	Trusting, “benefit of the doubt.”	Respect, understanding vs. resentment
Action Sequencing	Achieving a shared pattern of discussion across topics	Acceptance of discussion norms toward goal/decision	Mutual regard, patience vs. strain
